# Altered Co-Translational Processing Plays a Role in Huntington's Pathogenesis—A Hypothesis

**DOI:** 10.3389/fnmol.2016.00054

**Published:** 2016-07-06

**Authors:** Daniel A. Nissley, Edward P. O'Brien

**Affiliations:** O'Brien Lab, Department of Chemistry, The Pennsylvania State UniversityUniversity Park, PA, USA

**Keywords:** biophysics, Huntington's disease, kinetics, neurodegenerative disease, protein aggregation, translation, translation regulation, protein biogenesis

## Abstract

Huntington's disease (HD) is an autosomal dominant neurodegenerative disorder caused by the expansion of a CAG codon repeat region in the HTT gene's first exon that results in huntingtin protein aggregation and neuronal cell death. The development of therapeutic treatments for HD is hindered by the fact that while the etiology and symptoms of HD are understood, the molecular processes connecting this genotype to its phenotype remain unclear. Here, we propose the novel hypothesis that the perturbation of a co-translational process affects mutant huntingtin due to altered translation-elongation kinetics. These altered kinetics arise from the shift of a proline-induced translational pause site away from Htt's localization sequence due to the expansion of the CAG-repeat segment between the poly-proline and localization sequences. Motivation for this hypothesis comes from recent experiments in the field of protein biogenesis that illustrate the critical role that temporal coordination of co-translational processes plays in determining the function, localization, and fate of proteins in cells. We show that our hypothesis is consistent with various experimental observations concerning HD pathology, including the dependence of the age of symptom onset on CAG repeat number. Finally, we suggest three experiments to test our hypothesis.

## Introduction

Huntington's disease is an autosomal dominant neurodegenerative disorder characterized by the death of striatal neurons and the appearance of aggregates in the nuclei of a wide range of brain tissues (Davies et al., 1997; Tobin and Signer, 2000). Physical symptoms of HD include chorea (involuntary, dance-like motor function) and the dementia-like decline of mental faculties (Tobin and Signer, 2000). The genetic cause of HD is the expansion of a CAG codon repeat in Exon 1 of the HTT transcript; persons without HD have on average 19 CAG repeats, while individuals with 35 or more CAG repeats will develop HD symptoms over a typical lifespan (Squitieri et al., 1994; Brinkman et al., 1997; Lee et al., 2012). Each additional CAG repeat beyond 35 results in the onset of symptoms roughly 3 years sooner, with repeat lengths greater than 60 leading to acute juvenile onset (Brinkman et al., 1997; Li and Li, 1998; Lee et al., 2012).

The pathogenesis of HD is most likely due to one or more of the aberrant gain-of-function (Yano et al., 2014) or loss-of-function (Atwal et al., 2007) behaviors that have been identified for mutant huntingtin (mHtt) or the HTT transcript. Understanding the mechanism of pathogenesis is significantly complicated by the fact that the normal function of wild-type huntingtin protein (Htt) is not agreed upon (Cattaneo et al., 2005). Htt is composed of, starting from the N-terminus, a 17-amino acid localization signal (denoted N17) (Maiuri et al., 2013) that allows for the targeting of Htt to subcellular organelles including the Golgi apparatus, endoplasmic reticulum, and mitochondria (Rockabrand et al., 2007). Next in the sequence are 19 glutamines (Squitieri et al., 1994; Brinkman et al., 1997) (poly-glutamine) encoded by CAG codon repeats. Following the poly-glutamine region is a 38 amino acid proline-rich region (poly-proline) which contains a total of 28 prolines, including continuous stretches of 10 and 11 prolines (Crick et al., 2013). These three sequence elements of N17, the poly-glutamine region, and the poly-proline region, constitute Htt Exon 1 (Crick et al., 2013). The characteristic aggregates observed in HD patients are primarily constituted by small fragments of Htt Exon 1 that are most likely generated by abortive degradation attempts or the translation of an Exon 1-only mRNA produced due to aberrant splicing (Suhr et al., 2001; Chow et al., 2012; Sathasivam et al., 2013). Recent evidence suggests that C-terminal mHtt fragments can also lead to toxic effects by inhibiting the protein dynamin 1, leading to endoplasmic reticulum stress (El-Daher et al., 2015). The remainder of the 3144 residues in Htt consist of approximately 40 HEAT repeats, which are conserved helix-turn-helix structure motifs (Cattaneo et al., 2005) each roughly 40 residues in length. The only difference between the primary structure of mHtt and Htt, and the genetic cause of HD pathology, is that mHtt contains an expansion of the poly-glutamine region of Exon 1 to 35 or more glutamines.

Since the discovery in 1993 that HD is caused by CAG-repeat expansion in Htt Exon 1, no dominant hypothesis for HD pathogenesis has emerged. Instead, many interrelated hypotheses have been put forward that variously describe HD as the result of dysfunction at the protein (Truant et al., 2006) or mRNA (McLaughlin et al., 1996) level, to be associated with subcellular organelles like the mitochondria (Yano et al., 2014) or systems such as the ubiquitin-proteasome system (Chow et al., 2012), and to be due to either loss- or gain-of-function (Gil and Rego, 2008). Each of these hypotheses can explain some subset of the diverse set of experimental observations of HD pathogenesis and mHtt behavior, though the overall picture remains unclear. Despite this complex and overlapping set of hypotheses, new hypotheses continue to emerge that can explain more diverse, or previously disparate, sets of observations.

Here, we propose the novel hypothesis that co-translational processing plays a role in HD pathogenesis. This hypothesis is motivated by recent experimental results demonstrating that many of the behaviors of nascent proteins, including their targeting and aggregation, can be altered by changes to translation kinetics. We propose that a co-translational process is perturbed for mHtt due to altered translation-elongation kinetics downstream of N17. The magnitude of this perturbation is directly proportional to the increase in the length of the poly-glutamine sequence past Q_35_. Such perturbations to co-translational processes have been shown to profoundly alter downstream protein behavior, and the idea that there is a co-translational element to Huntington's pathogenesis is consistent with the experimental literature. We describe the insights gained from these studies and how they suggest a role for co-translational processes in HD pathology. We show that our hypothesis is consistent with a number of experimental observations concerning mHtt behavior and HD pathogenesis. We conclude by suggesting three experiments that directly test key aspects of our hypothesis.

## Co-translational processes influence post-translational protein behavior

A number of processes involving nascent proteins take place during protein synthesis. These processes are therefore referred to as co-translational processes, and include domain folding (Nicola et al., 1999; Komar, 2008), chaperone interactions (Gloge et al., 2014), translocation (Walter and Johnson, 1994; Pechmann et al., 2013), ubiquitination (Comyn et al., 2013; Duttler et al., 2013), phosphorylation (Oh et al., 2010; Keshwani et al., 2012), acetylation (Polevoda and Sherman, 2000), and glycosylation (Ruiz-Canada et al., 2009). The rates at which different nascent chain segments are synthesized can affect these co-translational processes, leading to altered nascent protein behavior in a cell (Zhang et al., 2009; Siller et al., 2010; Pechmann et al., 2014). These co-translational processes appear to occur far from equilibrium, such that the kinetics of translation can be more important than thermodynamics in determining nascent protein behavior (Nissley and O'Brien, 2014). Perturbations to these temporally-coordinated co-translational processes can result in deleterious downstream effects such as protein mistargeting (Kramer et al., 2009) and aggregation (Cortazzo et al., 2002). Most literature examples of codon-translation-rate-dependent phenomena are from prokaryotic organisms, such as *E. coli*. However, the timing of translation is also critical for nascent protein behavior in eukaryotic cells (Nissley and O'Brien, 2014). Eukaryotic cells contain homologs or molecules that carry out similar functions to those which act co-translationally in prokaryotes. Furthermore, the principles of non-equilibrium systems that underlie these phenomena are organism-independent (Nissley and O'Brien, 2014). For example, human cells contain a chaperone system homologous to the DnaJ/DnaK chaperone system that assists protein folding in prokaryotes.

### Nascent chain interactions with auxiliary factors depend on nascent chain length

Recent studies have shown that the interactions of ribosome nascent chain (RNC) complexes with targeting complexes (Noriega et al., 2014), enzymes (Sandikci et al., 2013), and chaperones (Rutkowska et al., 2008) are carefully orchestrated in cells, and that their equilibrium affinities for translationally-arrested RNCs depend sensitively on nascent chain length. For example, the signal recognition particle (SRP), a universally-conserved ribonucleoprotein that targets nascent proteins for translocation into the ER by selectively interacting with conserved signal sequences, interacts with RNCs in a nascent-chain-length-dependent manner (Noriega et al., 2014). Experiments have shown that SRP binds strongest to arrested-RNCs of between 75 and 95 amino acids in length; outside this region, the dissociation constant increases 3- to 24-fold (Noriega et al., 2014). Interactions between nascent chains and chaperones have also been shown to depend on nascent chain length. Trigger factor (TF) is a molecular chaperone in *E. coli* which assists the folding of nascent proteins by binding the ribosome during translation and shielding the nascent protein from aberrant interactions (Maier et al., 2005; O'Brien et al., 2012), helping to prevent misfolding and aggregation (Hoffmann et al., 2010). Similar to SRP, RNC/TF interactions are also optimal within a narrow range of nascent chain lengths, with a nearly 5-fold decrease in TF's dissociation constant for a RNC harboring a 100-residue nascent chain in comparison to a 23-residue nascent chain (Rutkowska et al., 2008). Nascent chain length is a key factor affecting these co-translational processes.

### Co-translational folding and downstream function depend on translation-elongation rates

During continuous translation *in vivo*, the dwell time of a ribosome at a given nascent chain length depends on the rate at which the codon in the A-site is decoded into an amino acid. The ribosome does not translate all codons at the same rate due to a variety of molecular factors (Fluitt et al., 2007; Stadler and Fire, 2011; Pop et al., 2014), and the variability in codon translation rates across an mRNA's coding sequence is a key parameter that modulates nascent protein behavior. Barral and co-workers found that “optimizing” the codon sequence of firefly luciferase (FL) by replacing rare codons (which are thought to translate more slowly than average) with common synonymous codons resulted in a ~55% decrease in specific activity *in vivo* (Spencer et al., 2012). In the case of the fast-translating FL transcript, the decrease in specific activity was accompanied by an increase in the amount of aggregated FL, suggesting that accelerating translation decreased FL's ability to acquire its correct structure and perform its intended function. Codon translation rates have also been shown to play a key role in regulating the structure and function of the *N. crassa* clock protein FRQ (Zhou et al., 2013). Optimization of the wild-type FRQ translation-rate profile resulted in the abolishment of *N. crassa*'s circadian rhythm and a two-fold decrease in FRQ's ability to interact with a binding partner, suggesting that changes to FRQ's translation-rate profile altered its structure and function.

### The co-translational targeting of nascent proteins depends on translation-elongation rates

Codon translation rates influence other co-translational processes in addition to nascent protein structure acquisition. The ability of SRP to target nascent chains for translocation depends not only on its equilibrium affinity for conserved N-terminal signal sequences measured on arrested-RNCs (Zhang and Shan, 2012), but also on the rate at which the signal sequence emerges from the exit tunnel during continuous synthesis in a cell. Globally decreasing codon translation rates increases the amount of protein that SRP successfully translocates into the ER (Zhang and Shan, 2012). A bioinformatic analysis (Pechmann et al., 2014) also revealed that “non-optimal” codons are systematically enriched in the genomes of nine yeast species 35–40 codons downstream of SRP signal sequences. The location of this downstream region would result in translational slowdown while the signal sequence is connected to the ribosome by a 35–40 amino acid linker, which corresponds to the approximate length of the ribosome exit tunnel (Yusupov et al., 2001), such that translation will be slowed just as the signal sequence emerges from the tunnel and SRP is sterically permitted to interact with it. This slowdown is presumed to give SRP more time to recognize and bind the signal sequence, ensuring that the nascent protein is successfully targeted to, and translocated into, the ER.

### Polyproline stretches slow-down translation elongation

The molecular origin of the observed variability of codon translation rates is complex, including factors such as cognate tRNA concentrations, the chemical nature of the amino acid being added to the nascent chain (i.e., the nature of the amino acid in the A-site), and sequence motifs within the nascent chain (Pavlov et al., 2008; Artieri and Fraser, 2014). For example, it has been well-established that poly-proline regions slow down translation. *In vitro* (Pavlov et al., 2008) and *in vivo* (Artieri and Fraser, 2014) experiments have demonstrated that the ribosome translates sequences of two or more prolines much slower than the average global translation rate.

The results we have discussed highlight how critical the timing of translation can be to co-translational phenomena and to determining downstream protein behavior in a cell.

## The hypothesis: Altered co-translational processes involving huntingtin play a role in HD pathology

**Figure 1:**
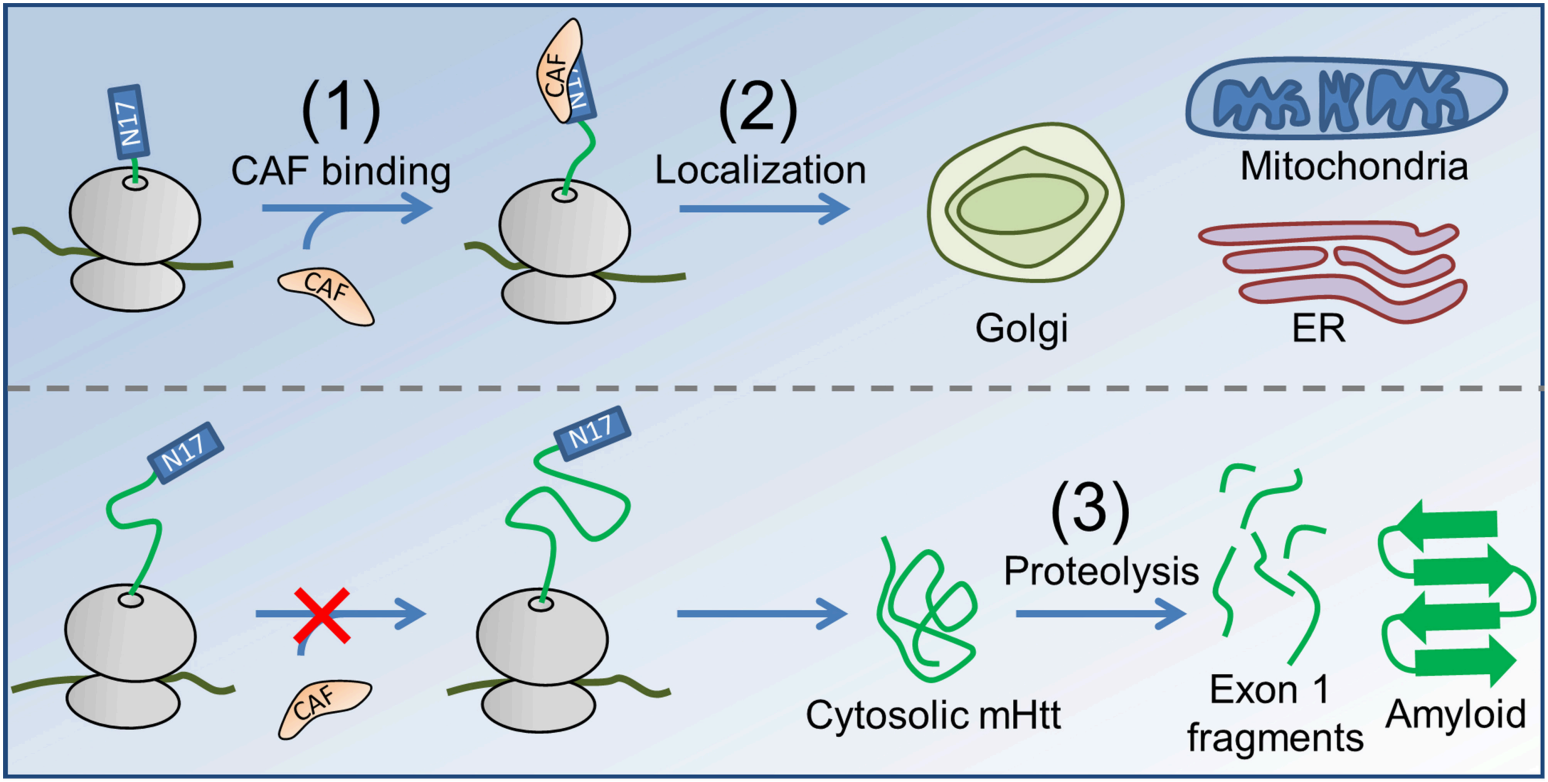
**The proposed co-translational mechanism of HD pathology**. (1) A CAF (orange) recognizes N17 (blue rectangle) of nascent Htt (top, nascent proteins shown in green, ribosome in gray). In the case of mHtt (bottom), the poly-proline region is not correctly positioned to slow translation as N17 emerges, reducing the ability of the CAF to bind. (2) In the case of Htt, the CAF directs the subcellular localization of Htt to the Golgi, ER, and mitochondria. Enzymatic modifications (not shown) may occur between targeting and localization, as is known to occur for some proteins in *E. coli* (Sandikci et al., 2013). mHtt is largely directed to the cytosol, where proteolysis (3) produces short, Exon 1-containing fragments (short green line segments) that form amyloid. Proteolysis can also result in C-terminal mHtt fragments that interfer with ER function (El-Daher et al., 2015).

Our hypothesis for the contribution of co-translational processes to HD pathogenesis naturally follows from these experimental observations of protein biogenesis. In Htt, stretches of prolines are optimally positioned 30–57 residues downstream of N17 to slow translation-elongation when N17 has just been exposed from the confines of the ribosome exit tunnel, which may also be the optimal length at which the binding between a co-translationally acting factor (CAF) and the nascent chain is strongest (Figure 1). These proline residues are highly conserved, being present in the huntingtin proteins of all higher vertebrates (Cattaneo et al., 2005). This slowdown of translation provides time for an as-yet-unidentified (and unlooked-for) CAF to interact with the nascent chain and either help direct it to its proper subcellular location or chemically modify the nascent chain as needed for its function. In the case of mHtt, however, the expanded poly-glutamine region, rather than the poly-proline region, will be undergoing translation as the N17 sequence emerges from the ribosome exit tunnel; translation of these glutamines (encoded by CAG) is two- to six-fold faster than translation of the prolines located at these same codon positions in the wild-type (Pavlov et al., 2008; Artieri and Fraser, 2014), and the CAF will thus have less time to bind N17 at the strongest-binding nascent chain lengths. A key concept in this hypothesis is that for each additional CAG repeat added to the poly-glutamine region there is a proportional decrease in the time available for the CAF to bind N17 at the nascent-chain lengths for which it has the strongest binding affinity at equilibrium. As a result, for each additional CAG repeat a smaller fraction of mHtt will interact with the CAF, and more mHtt will therefore have the opportunity to act aberrantly and form aggregates.

**Figure 2:**
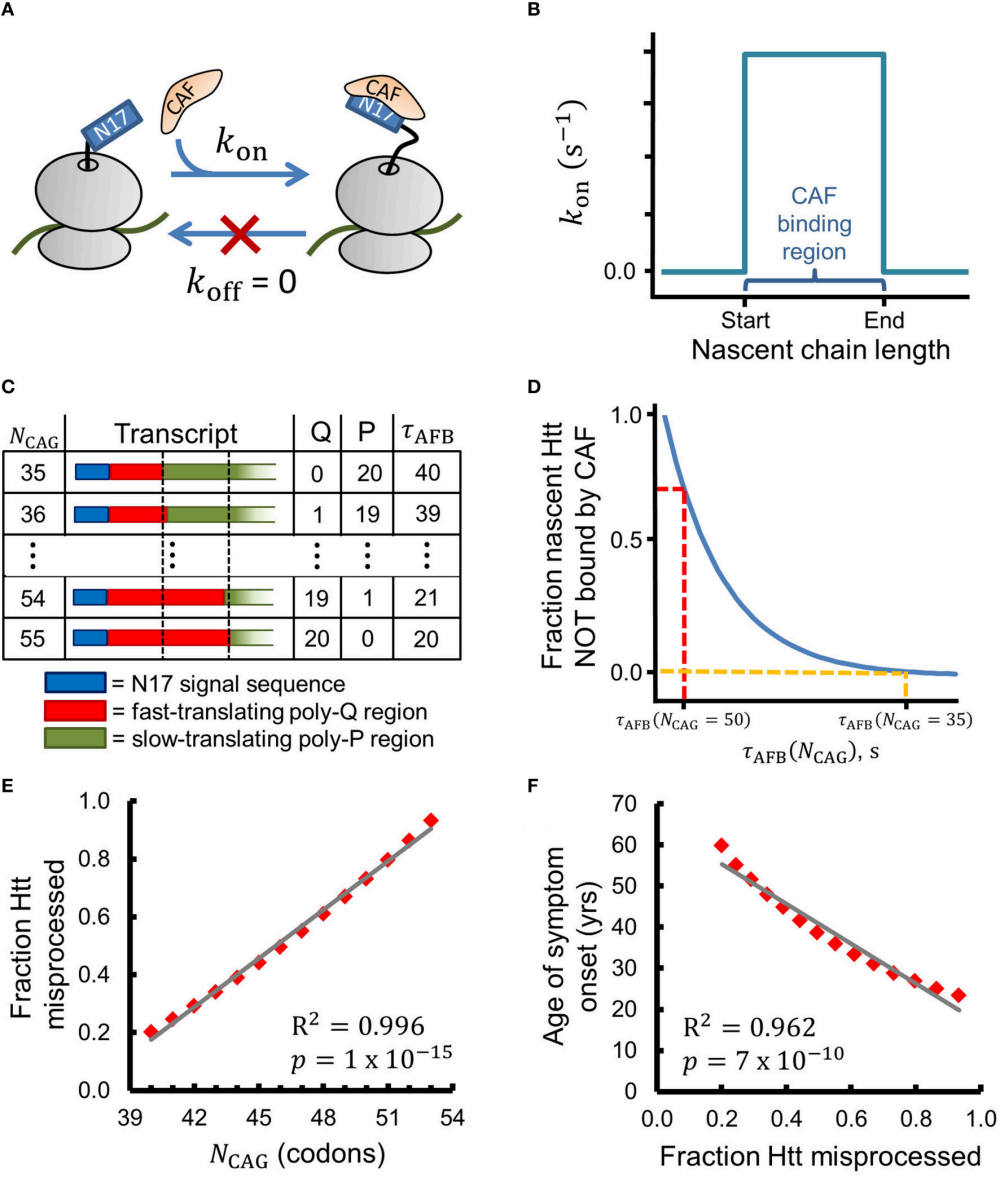
**A simple chemical-kinetic model explains how the age of onset of HD symptoms could arise from disruption of co-translational processing of huntingtin**. **(A)** The CAF is assumed to bind N17 irreversibly in the region of optimal binding with rate *k*_on_. **(B)** Within this model, the CAF can only bind when the nascent Htt molecule is between 52 and 71 amino acids in length (i.e., when the ribosome starts translating the poly-proline region). **(C)** As *N*_CAG_ increases the number of glutamines (Q) in the binding region increases and the number of prolines (P) decreases, leading to a decrease in the time available for CAF binding (τ_AFB_). τ_AFB_ has units of τ_A_ (see Methods). **(D)** The fraction of Htt that is co-translationally misprocessed depends on τ_AFB_ and, thereby, on *N*_CAG_, as expressed by Equation 1. **(E)** The fraction of Htt which is misprocessed (*f*_mp_ in Equation 1) strongly correlates with *N*_CAG_ when realistic values for *k*_on_ and CAF concentration are used (see Methods for a complete description of Equation 1). **(F)** The age of HD symptom onset shows strong negative correlation with the fraction of Htt misprocessed predicted by Equation 1. Age of onset vs. *N*_CAG_ data were extracted from Figure 1C of Lee et al. (2012) with PlotDigitizer (plotdigitizer.sourceforge.net).

This hypothesis is consistent with nine key Experimental Observations concerning HD and mHtt:

**Experimental Observation 1:** The brains of HD patients contain protein aggregates in cell nuclei (DiFiglia et al., 1997; Huang et al., 1999). The main constituents of these characteristic aggregates are fragments of mHtt Exon 1 (Suhr et al., 2001). In the cytosol, mHtt is targeted for degradation by either chaperone-mediated autophagy (Qi and Zhang, 2013) or the ubiquitin/proteasome system (UPS) (Chow et al., 2012).
**Explanation 1:** As the number of CAG repeats increases, more mHtt is co-translationally misprocessed and directed to the cytosol. The UPS' ability to clear mHtt decreases over time (Seo et al., 2004; Hunter et al., 2007), and it also has trouble completely degrading proteins with repetitive sequence elements like mHtt's poly-glutamine region (Fishbain et al., 2015). These problems with degradation combine with the increased partitioning of mHtt into the cytosol to increase the quantity of mHtt fragments in the cell, thereby making it more likely that they may enter the nucleus and aggregate or otherwise act aberrantly (Atwal et al., 2007). This increase in cytosolic mHtt may also lead to an increase in the quantity of C-terminal fragments that cause ER stress (El-Daher et al., 2015).
**Experimental Observation 2:** Only persons with 35 or more CAG repeats develop HD symptoms (Squitieri et al., 1994; Brinkman et al., 1997; Lee et al., 2012).
**Explanation 2**: If nascent Htt contains >35 CAG repeats, the poly-proline region is not positioned to slow translation as N17 emerges from the ribosome exit tunnel. Inefficient processing results due to decreased CAF binding, leading to the accumulation of mHtt in the cytosol and subsequent aggregation.
**Experimental Observation 3:** Each additional CAG repeat beyond 35 speeds disease progression by roughly 3 years (Squitieri et al., 1994; Brinkman et al., 1997; Lee et al., 2012).
**Explanation 3:** As the number of CAG repeats increases over 35, targeting becomes proportionally less efficient, impaired by the decreased time available for the CAF to bind mHtt's N17 sequence (see Figure 2). This decreased targeting efficiency results in an increased flux of mHtt into the cytosol, increasing the rate of aggregate formation.
**Experimental Observation 4:** N17 is required for the correct targeting of Htt to subcellular organelles (Rockabrand et al., 2007); the removal of N17 has been shown to completely abolish the targeting of Htt to the mitochondria, ER, and Golgi apparatus.
**Explanation 4:** CAFs can be sequence specific (Zhang and Shan, 2012), identifying motifs like conserved patterns of hydrophobic and charged residues (Xia et al., 2003). If N17 is absent, then there is nothing for the CAF to recognize and bind, resulting in the inefficient targeting of mHtt to subcellular organelles (Rockabrand et al., 2007).
**Experimental Observation 5**: Removal of N17 leads to a large increase in the rate of nuclear aggregate formation in a HD mouse model, despite lower expression levels (Gu et al., 2015).
**Explanation 5**: Removal of N17 effectively abolishes targeting of nascent Htt to subcellular organelles (see Experimental Observation 4). The ΔN17 deletion mutant of mHtt therefore remains in the cytosol for an extended period of time, further burdening the UPS and speeding the formation of the Exon 1 fragments that form nuclear aggregates or of the C-terminal fragments that induce ER stress (Suhr et al., 2001; El-Daher et al., 2015).
**Experimental Observation 6:** The length of the poly-glutamine region alters Htt targeting (Rockabrand et al., 2007). Increasing the length of the poly-glutamine domain from 25 to 97 Q's reduces co-localization of the protein to the mitochondria, ER, and Golgi by 4, 10, and 30%, respectively.
**Explanation 6:** Expansion of the poly-glutamine sequence moves the poly-proline stretch further downstream, disrupting the wild-type translation-elongation schedule. Without the poly-proline region to slow translation at the proper time, N17 is less likely to correctly interact with a CAF, in turn reducing the amount of correctly localized mHtt.
**Experimental Observation 7:** The presence of the poly-proline region is critical for correct targeting (Rockabrand et al., 2007). Removal of the poly-proline region reduces the co-localization of the protein to the ER and Golgi by 30 and 25%, respectively.
**Explanation 7:** Complete removal of the poly-proline region significantly perturbs the wild-type translation-rate profile of Htt. There will be less time available for a CAF to interact with N17, decreasing the probability of correct targeting and downstream function.
**Experimental Observation 8**: Removal of the poly-proline region is detrimental to spatial learning and memory in a mouse model of HD (Neveklovska et al., 2012).
**Explanantion 8**: As described in Experimental Observation 7, removal of the poly-proline region leads to an increase in the quantity of Htt which is unable to perform its correct downstream function, leading to the observed disease symptoms.
**Experimental Observation 9:** A fusion protein consisting of the first 171 amino acids of Q125 mHtt attached to GFP has a shorter soluble half-life than an analogous Htt171-GFP fusion protein (Kaytor et al., 2004).
**Explanation 9:** This decreased solubility is due to perturbed co-translational processing, which can result in poor targeting to subcellular organelles that may increase aggregation propensity and result in a decreased soluble half life (Cortazzo et al., 2002).

Though other hypotheses for HD pathology can explain different subsets of these observations, our hypothesis is the only one (Zuccato et al., 2003; Cornett et al., 2005; Atwal et al., 2007; Nalavade et al., 2013; Yano et al., 2014) that, to our knowledge, is consistent with the experimentally-observed dependence of proper Htt targeting on the presence of the N17, poly-glutamine, and poly-proline sequence motifs. Our hypothesis is also unique in offering a molecular explanation for why removal of the N17 or poly-proline sequences leads to the development of disease symptoms.

## Our hypothesis in the context of other mechanisms that could contribute to mHtt pathology

Many hypotheses have been proposed (McLaughlin et al., 1996; Zuccato et al., 2003; Cornett et al., 2005; Atwal et al., 2007; Rockabrand et al., 2007; Chow et al., 2012; Nalavade et al., 2013; Yano et al., 2014) (a partial list) that can explain different groupings of the nine experimental observations of HD pathogenesis and Htt behavior we list above. We cannot succinctly describe the full complexity of these interrelated theories. However, given the complexity of HD pathogenesis, it seems reasonable that each of these mechanisms has the potential to contribute in some way to the disease phenotype.

None of the other hypotheses that offer explanations for Experimental Observations 1–9 are mutually exclusive with the model that we have described. For example, the hypothesis that mHtt toxicity is mRNA-mediated (McLaughlin et al., 1996; Nalavade et al., 2013) posits that large CAG-repeat lengths in HTT can cause toxic downstream effects by forming a stable mRNA hairpin that sequesters a diverse set of proteins. Our co-translational hypothesis of HD pathogenesis can co-exist with this hypothesis. Consider one implication of the RNA-mediated hypothesis: at high values of *N*_CAG_ a hairpin can form in HTT that recruits various cellular factors, including protein kinase R (PKR) (Peel, 2004). PKR is a double-stranded RNA-dependent kinase that forms part of the cellular virus-defense system, and its activation is associated with myriad downstream cell stress and apoptosis events including the upregulation of proteolysis machinery (Peel, 2004). Simultaneously, perturbation of co-translational processes may increase the fraction of mHtt directed to the cytosol (Figure 1), such that both processes synergistically contribute to an increase in the quantity of mHtt fragments observed *in vivo*. This explanation also demonstrates the interplay between our hypothesis and the hypothesis that proteolysis of mHtt is key to pathogenesis, as perturbed co-translational interactions that increase the amount of mHtt directed to the cytosol would also increase the stress on the cell's proteolysis machinery (Chow et al., 2012).

Repeat-associated non-ATG translation initiation of the mHtt transcript may also be related to our co-translational hypothesis. The frameshift created by this non-canonical translation initiation process will alter the identity of the codons being translated and also result in a nascent protein without the N17 localization sequence (Nalavade et al., 2013). These two consequences of non-ATG translation initiation constitute a significant alteration to codon elongation rates and co-translational behavior that may contribute to HD pathology. Consider also that our hypothesis is independent of the method of mHtt fragment generation. Whether pathogenic fragments are a result of proteolysis or of aberrant mRNA splicing of Exon1, translation will still occur, and co-translational phenomena can thus still be critical. Experiments have also shown that the MID1-PP2A regulatory complex binds the mRNA hairpin formed by the expanded CAG-repeat region of mutant HTT, increasing the rate of translation initiation (Krauss et al., 2013). This increase in translation initiation for the mutant transcript increases the amount of mHtt translated, increasing the amount of protein that may be co-translationally misprocessed according to our hypothesis.

As previously mentioned, ours is the only hypothesis, to our knowledge, that can explain the experimentally-observed influence of the N17, poly-glutamine, and poly-proline regions on the sub-cellular localization of Htt and mHtt, the large increase in nuclear aggregation when N17 is deleted, and the relationship between poly-proline deletion and various negative effects (Experimental Observations 4–8). The ability of our hypothesis to explain the large increase in aggregation rate observed when mHtt lacking N17 is expressed *in vivo* (Experimental Observation 5) is particularly important. *In vitro* studies have demonstrated that the aggregation rate of purified poly-glutamine segments increases as the number of poly-glutamine residues increases (Scherzinger et al., 1999). Occam's Razor suggests, then, that our co-translational phenomenon based hypothesis may be superfluous. However, this simpler hypothesis cannot explain the observed increase in aggregation *in vivo* upon N17 deletion, whereas our hypothesis can.

## A kinetic model based on this hypothesis suggests why the age of onset of HD symptoms negatively correlates with the number of CAG repeats

We can express this hypothesis in terms of the chemical reaction scheme shown in Figure 2A, in which we assume that a CAF only binds nascent Htt in a narrow range of nascent chain lengths with rate *k*_on_. This reaction scheme can be solved analytically using the principles of chemical kinetics to give an equation that estimates the change in the fraction of misprocessed nascent mHtt (*f*_mp_) as the number of CAG repeats (*N*_CAG_) increases above 35. By misprocessed we mean mHtt fails to interact with the CAF; for the purposes of this simple model, we assume that this co-translational interaction is an obligate step in Htt's normal protein maturation pathway. To derive this equation we make the simplifying assumptions that (i) the binding of the hypothesized CAF to N17 is irreversible in the region of optimal binding, (ii) *k*_on_ is non-zero from codon position 53, when translation of the poly-proline region begins for Q35-Htt, to codon position 72 (corresponding to nascent chain lengths 52–71, see Figure 2B and Methods), and (iii) prolines are translated twice as slowly as glutamines (Figure 2C and Methods). Assumptions (ii) and (iii) have experimental support in the literature, as described in the Methods. With these assumptions, *f*_mp_ (see Methods for a complete description of this kinetic model and its technical assumptions), which calculates the relative fraction of Htt misprocessed at *N*_CAG_ in comparison to the amount misprocessed when *N*_CAG_ = 35, can be expressed as

(1) $$\begin{array}{r} f_{\text{mp}}\left(N_{\text{CAG}}\right)=\text{exp}\left[-k_{\text{on}}\left(\tau_{\text{AFB}}\left(N_{\text{CAG}}\right)-\tau_{\text{AFB}}\left(35\right)\right)\right]-1. \end{array}$$

In Equation (1), τ_AFB_ is the total amount of time available for CAF binding at the optimal nascent-chain binding lengths, which is a function of *N*_CAG_ (Figure 2C). As τ_AFB_ decreases the fraction of misprocessed Htt increases (Figure 2D). Figure 2E displays the results obtained from Equation (1) when a realistic value for *k*_on_ (see Methods) is used to calculate *f*_mp_ for values of *N*_CAG_ from 40 to 53. We find that *f*_mp_ strongly correlates with $N_{\text{CAG}}\left({\text{Pearson R}}^{2}=0.996,p=1\times 10^{-15}\right)$. Furthermore, we find that the experimentally-determined age of HD symptom onset also strongly correlates with *f*_mp_ (PearsonR^2^ = 0.962, *p* = 7 × 10^−10^, Figure 2F). This latter correlation is consistent with our hypothesis that a defect in a co-translational process plays a role in HD pathogenesis. Strong correlations are also found between our kinetic model and the age of onset data presented by Brinkman et al. (1997) for *N*_CAG_ values of 39–50 (*f*_mp_ vs. *N*_CAG_: Pearson R^2^ = 0.997, *p* = 7 × 10^−14^; Age of onset vs. *f*_mp_: Pearson R^2^ = 0.958, *p* = 3 × 10^−8^, data not shown) (Brinkman et al., 1997).

## On the nature of misprocessing

With over 11 different co-translational processes potentially acting on huntingtin, any one or more of them may be perturbed by altered translation-elongation kinetics due to CAG expansion. We believe, however, that the two most-likely culprits are (i) altered co-translational phosphorylation of N17 by a CAF or (ii) decreased binding of a CAF that targets Htt to its proper cellular location.

Consider, for example, the influence that CAG-repeat expansion could have on the phosphorylation of Htt at serine positions S13 and S16 within N17 and, thereby, on downstream Htt behavior such as membrane binding. Experiments have demonstrated that the phosphorylation of these two serine residues is important for the cellular localization of Htt (Atwal et al., 2011; Maiuri et al., 2013) and also plays a role in determining if Htt will be targeted for degradation by the ubiquitin/proteasome system (UPS) (Thompson et al., 2009). Critically, mHtt has also been experimentally shown to have decreased levels of phosphorylation in comparison to Htt (Atwal et al., 2011). Such changes in phosphorylation state have been shown to influence the binding affinity of peptides for membranes (Dehlin et al., 2008). Our hypothesis can succinctly explain these observations. The N-terminal location of S13 and S16 within huntingtin means that the time available for co-translational phosphorylation will decrease as the number of CAG repeats increases (see Figures 2C,D). In the case of Htt, the poly-proline sequence is correctly placed to slow translation as N17 emerges, allowing time for the phosphorylation of the serines and subsequent downstream localization and function of Htt. In the case of mHtt, however, co-translational phosphorylation is perturbed by the decrease in time available for CAF binding introduced by the expansion of the CAG-repeat region, resulting in a decrease in the fraction of mHtt which is correctly phosphorylated and can perform its correct downstream function. An analogous mechanism can be postulated for each of the N-terminal processing events which may affect Htt co-translationally, such as N-terminal acetylation and phosphorylation of Thr3 (Aiken et al., 2009). It is important to note that this example assumes N17 phosphorylation occurs co-translationally; while there is evidence that this is the case for some proteins (Oh et al., 2010; Keshwani et al., 2012), whether or not Htt is co-translationally phosphorylated has not yet been investigated.

## Testing the co-translational misprocessing hypothesis of HD pathology

Though our hypothesis is consistent with a wide range of experimental observations concerning HD pathogenesis, it is based on a large body of circumstantial evidence from the co-translational folding field. We therefore suggest three experiments that would test this model. First and foremost, it must be determined if a CAF engages Htt. This question can be answered with a combination of pulse-chase labeling and cross-linking assays. Pulse-chase assays have been used extensively to study co-translational protein folding, as they provide the ability to specifically visualize nascent proteins (Braakman et al., 1991; Nicola et al., 1999), while chemical crosslinking has also been used to capture RNC/CAF complexes *in situ* (Oh et al., 2011). We suggest that these two techniques be combined into a hybrid technique. A short pulse-period of radiolabel incorporation (<1 min) would be followed by a chase period with unlabeled media containing a crosslinking agent (such as dithiobis succinimidyl propionate Oh et al., 2011) to selectively label nascent Htt and capture its interactions with any CAFs, respectively. Similar methods utilizing the incorporation of non-canonical amino acids into Htt followed by photo-crosslinking (Mackinnon et al., 2007) or click chemistry (Dieterich et al., 2007) to connect Htt to binding partners can also be envisioned, though these methods require chemical modification of N17 that may alter the ability of potential binding partners to recognize and bind Htt Exon 1 (Maiuri et al., 2013). The experiment would conclude with size-dependent separation by SDS-PAGE and gel visualization *via* phosphorimaging or with mass spectrometry, depending on the technique used. Pending the positive identification of a Htt/CAF interaction, the next relevant question is whether this CAF preferentially binds Htt over mHtt. This question can be straightforwardly answered *via* the application of a number of different experimental techniques for determining dissociation constants as well as the on and off rates of RNC/CAF binding (Rutkowska et al., 2008).

Our hypothesis suggests that the amount of mHtt directed to the cytosol (i.e., the amount of nascent protein that is mistargeted or otherwise malfunctions and is available for proteolysis) is greater than the amount of Htt directed to the cytosol. Furthermore, our hypothesis predicts that the amount of mHtt directed into the cytosol will be a monotonically-increasing function of poly-glutamine length. Though the subcellular localization of various Htt Exon 1 constructs has been previously reported (Rockabrand et al., 2007), these data are not sufficient to quantify the flux of Htt/mHtt into the cytosol. Similar experiments could be designed utilizing separate fluorescent tags for each relevant organelle (i.e., the Golgi, ER, and mitochondria) as well as for the Htt construct. The fraction of cytosolic protein at various times after the start of the experiment could then be calculated by the difference between the total Htt-associated fluorescence and the Htt-associated fluorescence that co-localized with subcellular organelles.

## Conclusion

We have described a novel hypothesis that presents possible contributions to HD pathology due to perturbation of the non-equilibrium phenomena of co-translational nascent-protein processing. Within this model, N17 is the CAF binding site, the poly-glutamine region acts as a linker connecting N17 to the poly-proline region, and the poly-proline stretch acts as a brake on translation elongation that facilitates N17-CAF interactions. As the number of CAG repeats increases above 35, the poly-proline stall site shifts further and further downstream of N17, and due to the decreased time available for CAF binding, more mHtt fails to be correctly co-translationally modified or targeted and is therefore directed to the cytosol. In the cytosol, proteolysis can result in the production of Exon 1 or C-terminal fragments that form HD's characteristic nuclear amyloid (Suhr et al., 2001; Chow et al., 2012) or cause ER dysfunction (El-Daher et al., 2015), respectively. There is precedence for such an effect for other proteins; codon translation rates have been implicated as causal factors in the development of some human cancers (Supek et al., 2014; Zheng et al., 2014), cystic fibrosis (Bartoszewski et al., 2010), and disparate drug transport functionalities between synonymous mutant proteins (Kimchi-Sarfaty et al., 2007). Our hypothesis is consistent with key observations of mHtt behavior and HD pathology (Figures 1, 2). Furthermore, our hypothesis is testable, and we have suggested a number of experiments that do so.

Pending the identification of a CAF that interacts with mHtt new therapeutic strategies can be explored based on the idea of reducing the rate of poly-glutamine translation to provide the needed time for CAF binding. Though we have thoroughly investigated our co-translational hypothesis only for HD, five other poly-glutamine proteins associated with the neurodegenerative disorders SBMA, DRPLA, SCA-2, SCA-3, and SCA-7 also contain poly-proline regions (The Uniprot Consortium, 2015). Therefore, it is possible that these other proteins might also have a contribution to their pathology due to changes to co-translational phenomena upon poly-glutamine expansion. However, given the differences between these other poly-glutamine proteins and Htt, the specific form of this effect is likely different. It is our hope that the novel perspective offered in this paper will motivate experimentalists to further explore the molecular biophysics of HD pathology and any connection to translation kinetics and nascent protein behavior.

## Methods

### Derivation of chemical-kinetic model for Htt misprocessing

CAF binding tends to be favored in a narrow range of nascent chain lengths. For example, equilibrium binding data show that SRP's affinity for arrested RNCs is optimal within a ~20 amino-acid region (Noriega et al., 2014). Outside this optimal region, the *K*_D_ increases by 3- to 24-fold. Therefore, in our chemical kinetic reaction scheme (Figures 2A,B) we allow CAF/N17 binding only in a narrow range of nascent chain lengths by assuming that *k*_on_ is zero except between codon positions 53–72. We also assume that *k*_off_ is equal to zero, which approximates a binding process that heavily favors association over dissociation in the region of optimal binding. The ribosome first encounters the codons encoding the poly-proline region at codon 53 in Htt with a 35-residue poly-glutamine region. We therefore take position 53, somewhat arbitrarily, as the start of the CAF binding region (Figure 2B), which extends to codon position 72. Increasing the width of this optimal-binding region does not influence the results obtained in the calculation of Equation (1) (data not shown). In reality, the nascent chain length regime over which a CAF prefers to engage nascent Htt may be significantly different than that which we use here. Under these assumptions the amount of misprocessed Htt (*A*_mp_) can be expressed as a function of the total time available for CAF binding (τ_AFB_, the dwell time of the RNC in the optimal-binding region). These ideas are expressed mathematically in Equation (2).

(2) $$\begin{array}{rcl} A_{\text{mp}}\left(\tau_{\text{AFB}}\left(N_{\text{CAG}}\right)\right) & = & A_{\text{mp}}\left(\tau_{\text{AFB}}=0\right)*\\ \text{exp}\left(-k_{\text{on}}\tau_{\text{AFB}}\left(N_{\text{CAG}}\right)\right) \end{array}$$

*A*_mp_(τ_AFB_ = 0) is the amount of Htt misprocessed when there is no time available for CAF binding. The value of τ_AFB_ depends on *N*_CAG_ and is the total time required by the ribosome to decode codons *i* = 53 to *i* = 72 (Figure 2C). For simplicity, we assume that there are two types of codons, faster-translating codons, which are decoded in time τ_*A*_, and slower-translating codons, which are decoded in time 2τ_*A*_ (Figure 2C). Codons in the poly-glutamine region are defined to be faster-translating, while codons in the poly-proline region are defined to be slower-translating. Thus, τ_AFB_ decreases by an increment of τ_A_ for each CAG repeat past 35 (Figure 2C). Experimental reports suggest that proline codons require between two and six times longer to translate than the global average codon translation time (Pavlov et al., 2008; Artieri and Fraser, 2014). Figures 2E,F display the results when we use the smallest difference suggested by the literature of a two-fold increase in translation time of prolines. Equation 1 gives strong correlations when translation times between 2τ_*A*_ and 6τ_*A*_ are used (data not shown). As *N*_CAG_ increases over 35 and τ_AFB_ decreases, the fraction of nascent Htt which is *incorrectly* processed increases (Figure 2D). In order to avoid the issue of estimating a value for *A*_mp_(τ_AFB_ = 0), we consider instead the fraction of Htt which is misprocessed (*f*_mp_) at *N*_CAG_ relative to the amount of Htt misprocessed at *N*_CAG_ = 35,

(3) $$f_{\text{mp}}(N_{\text{CAG}})=\frac{\begin{array}{l} A_{\text{mp}}\left(\tau_{\text{AFB}}=0\right)[\text{exp}\left(-k_{\text{on}}\tau_{\text{AFB}}\left(N_{CAG}\right)\right)\\ \;-\text{exp}\left(-k_{\text{on}}\tau_{\text{AFB}}\left(35\right)\right)] \end{array}}{A_{\text{mp}}\left(\tau_{\text{AFB}}=0\right)\text{exp}\left(-k_{\text{on}}\tau_{\text{AFB}}\left(35\right)\right)}.$$

Algebraic simplification of Equation (3) yields Equation (1), which gives the fraction of misprocessed Htt as a function of *N*_CAG_ and does not depend on *A*_mp_(τ_AFB_ = 0). Equation (1) is linear for small arguments of the exponential term. Consider the power series expansion of exp(*x*),

(4) $$\begin{array}{l} \text{exp}\left(x\right)=\overset{\infty }{\underset{n=0}{\sum }}\frac{x^{n}}{n!}=1+x+\frac{x^{2}}{2!}+\frac{x^{3}}{3!}+\ldots \end{array}$$

For values of *x* ≪ 1, the *n* > 1 terms in Equation 4 are vanishingly small, and exp(*x*) is reasonably linear. We also note that altering the width and/or location of the optimal-binding region will alter the range of *N*_CAG_ values over which *f*_mp_ is a monotonically-increasing function. Once *N*_CAG_ increases such that all codons in the optimal-binding region encode Q and not P, τ_AFB_ is minimized and *f*_mp_ remains constant. With the sample numbers used here, τ_AFB_ reaches a minimum value of 20 at *N*_CAG_ = 55 (see Figure 2C), such that *f*_mp_becomes constant when *N*_*CAG*_ ≥ 55.

### Calculation of *k*_on_ for the chemical kinetic model

Equation (1) requires a rate constant for CAF/RNC binding in order to predict *f*_mp_. Binding rates are available in the literature for several different CAFs, including trigger factor, signal recognition particle, DnaK, and DnaJ. The rates reported in the literature typically have units of M^−1^s^−1^, indicating a dependence on both the cellular concentration of the CAF and time. First, we selected the *k*_on_ rate measured for DnaJ of 3.3 × 10^5^ M^−1^s^−1^ (Powers et al., 2012). Next, we determined a reasonable estimate for the intracellular concentration of DnaJ in human cells. Finka and Goloubinoff (2013) recently reported intracellular concentrations for 147 molecular chaperones in HeLa cells. We generated a reasonable estimate of the DnaJ intracellular concentration by taking the median of the cellular concentrations of the subset of 109 chaperones which were identified to be cytosolic or nuclear. This median value is 1.11 × 10^−7^ M (assuming an average HeLa cell volume of 2600 μm^3^ Finka and Goloubinoff, 2013). Multiplying the on rate for DnaJ by this concentration yields the *in vivo* *k*_on_ estimate used in Eq. 1 of 0.0366 s^−1^.

## Author contributions

EO first proposed the hypothesis. DN and EO conducted the research and wrote the manuscript.

## Funding

This project was supported by a Human Frontiers in Science Foundation program grant (RGP0038/2015).

### Conflict of interest statement

The authors declare that the research was conducted in the absence of any commercial or financial relationships that could be construed as a potential conflict of interest.
